# The HD-ZIP I transcription factor BcLMI1 regulates bolting time in *Brassica campestris* by repressing *BcTCP4*

**DOI:** 10.1093/hr/uhag157

**Published:** 2026-04-17

**Authors:** Jingwen Chen, Yujie Wu, Airong Li, Yuan Tian, Li Huang

**Affiliations:** Zhejiang University College of Agriculture and Biotechnology, Laboratory of Cell & Molecular Biology, Institute of Vegetable Science, Zhejiang University, Hangzhou 310058, China; Zhejiang University College of Agriculture and Biotechnology, Laboratory of Cell & Molecular Biology, Institute of Vegetable Science, Zhejiang University, Hangzhou 310058, China; Hainan Institute of Zhejiang University, Sanya 572025, China; Zhejiang University College of Agriculture and Biotechnology, Laboratory of Cell & Molecular Biology, Institute of Vegetable Science, Zhejiang University, Hangzhou 310058, China; Zhejiang University College of Agriculture and Biotechnology, Laboratory of Cell & Molecular Biology, Institute of Vegetable Science, Zhejiang University, Hangzhou 310058, China; Zhejiang University College of Agriculture and Biotechnology, Laboratory of Cell & Molecular Biology, Institute of Vegetable Science, Zhejiang University, Hangzhou 310058, China

## Abstract

During the domestication of *Brassica campestris* (syn. *Brassica rapa*), significant variations in bolting time have emerged, critically impacting crop yield and quality. To elucidate the genetic basis of bolting time regulation in *B. campestris*, we integrated selective-sweep analysis on 365 resequenced accessions with genome-wide association studies. This identified genes including *BcLMI1-LF* (*LATE MERISTEM IDENTITY 1*), an HD-ZIP I subfamily gene in the least fractionated subgenome of *B. campestris*. Further, gene ontology enrichment analysis indicated that *BcLMI1-LF* was a key candidate regulator of bolting.‌ Haplotype analysis demonstrated the association of Hap3 of *BcLMI1-LF* with the late bolting phenotype. Functional validation confirmed that *BcLMI1-LF-Hap3* overexpression significantly delays bolting. Mechanistically, yeast one-hybrid (Y1H), Dual-Luciferase Reporter Assay (DLRA), and chromatin immunoprecipitation followed by quantitative polymerase chain reaction (ChIP-qPCR) assays established that BcLMI1-LF-Hap3 directly represses *BcTCP4-LF* (*TEOSINTE BRANCHED 1*/*CYCLOIDEA*/*PCF 4*), a conserved bolting regulator.‌ ‌This study advances understanding of bolting regulation in *B. campestris* and provides molecular targets for breeding late-bolting crops.

## Introduction

*Brassica campestris* (syn. *Brassica rapa*), a widely cultivated cruciferous crop in Asia, predominantly produces edible leaves and leafy heads [1]. Consequently, premature bolting, the untimely transition from vegetative to reproductive growth, frequently leads to reduced yield and compromised quality. Bolting time is influenced by multiple factors, including photoperiod [2, 3], temperature [2, 4], and endogenous hormones [5, 6]; once initiated, leaf production ceases, resulting in rapid deterioration of commercially important traits [7, 8]. Therefore, elucidating the genetic basis and regulatory mechanisms of bolting is crucial for *B. campestris* breeding, especially in developing late-bolting cultivars adapted to changing environments.

The homeodomain-leucine zipper (HD-ZIP) transcription factor (TF) family, unique to plants, plays pivotal roles in development through its conserved HD for DNA binding and ZIP motif for protein dimerization [9]. This family orchestrates key developmental processes, such as shoot apical meristem maintenance [10], leaf polarity determination [11], vascular patterning [12], and stress adaptation [13], highlighting its functional versatility in plant growth and environmental responses.

Based on sequence phylogeny, domain architecture, and functional characterization primarily from *Arabidopsis thaliana*, HD-ZIP proteins are categorized into four distinct subfamilies (I, II, III, and IV) [14, 15]. LATE MERISTEM IDENTITY 1 (LMI1), a member of subfamily I, is a key regulator of plant development [16]. In *A. thaliana*, LMI1 directly modulates meristem identity and floral transition by influencing genes, such as *LEAFY* (*LFY*) and *AGAMOUS-LIKE 24* [17]. Notably, in Brassica crops (e.g. *B. rapa*, *B. oleracea*, *B. napus,* and *B. juncea*), allelic variations in LMI1 are associated with leaf margin morphology changes, suggesting its role in domestication [18–22]. However, whether LMI1 regulates bolting time or contributes to historical selection during *B. campestris* domestication remains unresolved.

Another key regulator of bolting time discovered recently is TEOSINTE BRANCHED 1/CYCLOIDEA/PCF 4 (TCP4), a plant-specific TF that integrates environmental and developmental cues. In *A. thaliana*, TCP4 directly activates *CONSTANS* (*CO*) and synergizes with *GIGANTEA* (*GI*) to mediate photoperiod-dependent flowering [23]. Post-transcriptional control by microRNA319 (miR319) further links *TCP4* to leaf development and hypocotyl elongation [24]. Strikingly, in *Lactuca sativa*, *LsTCP4* co-localizes with loci governing both leaf morphology and bolting time [25], underscoring its pleiotropic function. Whether TCP4 interacts with other TFs, such as LMI1, to coordinately control bolting remains unknown.

In this study, we employed an integrated approach combining population genomics and functional validation to elucidate the genetic control of bolting time. Our findings uncover a novel regulatory relationship underlying bolting time domestication in *B. campestris*, where *BcLMI1*, as a triplicated HD-ZIP I gene retained after whole-genome triplication, acts as a key repressor of *BcTCP4-LF* that governs bolting time.

## Results

### Key genes regulating bolting time in *B. campestris* domestication

**Figure 1:**
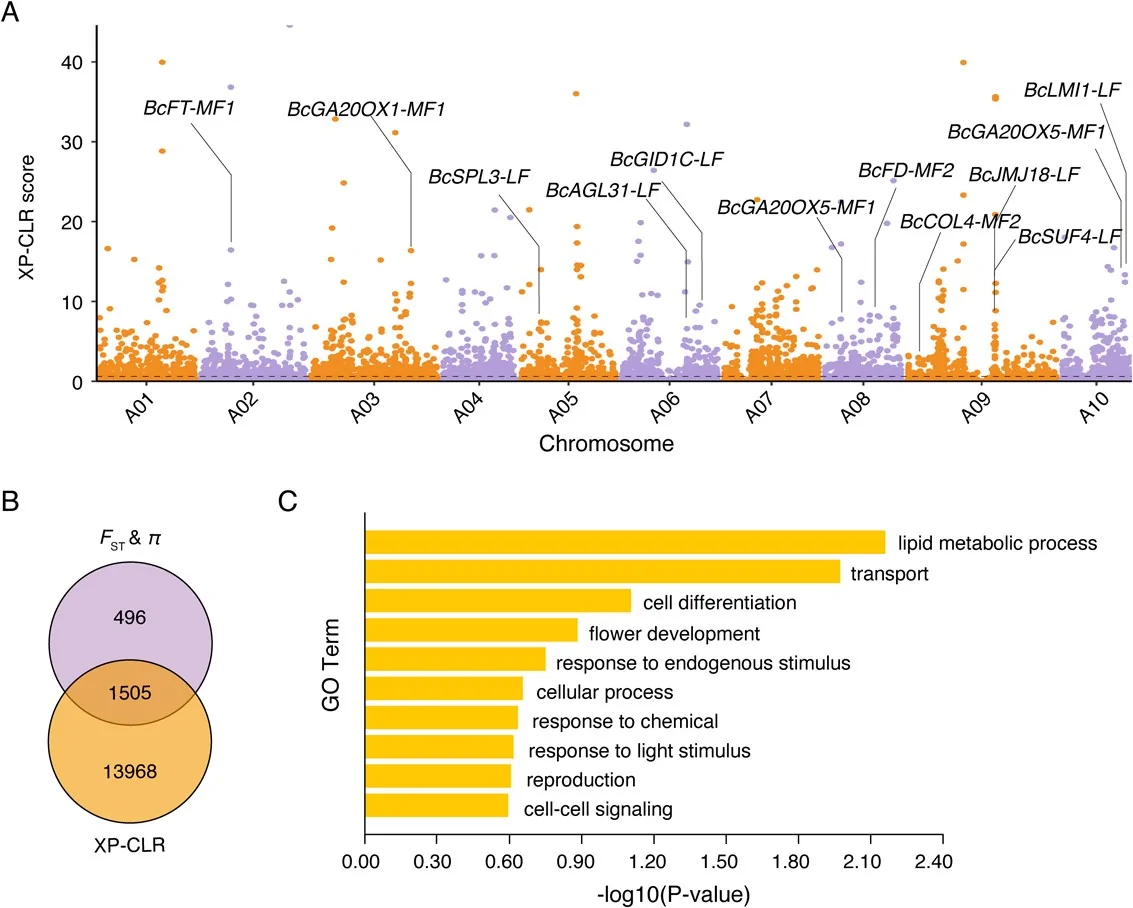
Domestication of bolting time in *B. campestris*. (A) Manhattan plot of XP-CLR scores across the ten chromosomes (A01–A10). Candidate genes with strong selection signals for bolting time are labeled. (B) Venn diagram showing the overlap of genes in selected regions identified by combining *F*_ST_, π, and XP-CLR analysis. (C) GO enrichment analysis of the 1505 candidate genes.

During the domestication of *B. campestris*, substantial variation in bolting time has emerged. To identify genomic regions associated with bolting, we categorized 365 resequenced accessions into early- and late-bolting groups, conducting a selective-sweep analysis. A total of 725 regions containing 2001 genes were identified by combining the results of *F*_ST_ and *π* analyses (Fig. S1; Tables S1–S3), while 2937 regions with 15 473 genes were detected using the Cross-Population Composite Likelihood Ratio (XP-CLR) method (Fig. 1A; Tables S4 and S5). By intersecting the results from all three methods, 1505 overlapping genes were identified within the selective regions (Fig. 1B). Gene ontology (GO) enrichment analysis of these genes revealed biological processes associated with bolting control, such as ‘flower development’, which contain 28 genes (Fig. 1C; Table S6). The candidate genes associated with these GO terms include core bolting integrators such as *FLOWERING LOCUS T* (*BcFT-MF1*), *FD* (*BcFD-MF2*), and *SQUAMOSA PROMOTER BINDING PROTEIN-LIKE 3* (*BcSPL3-LF*). Additionally, several genes involved in the gibberellin (GA) pathway were identified, including *GIBBERELLIN 20-OXIDASE 3* (*BcGA20OX3-LF*), *BcGA20OX1-MF1*, *BcGA20OX5-MF1*, and the GA receptor *GA INSENSITIVE DWARF1C* (*BcGID1C-LF*). Other key regulators associated with bolting time include *AGAMOUS-LIKE 31* (*BcAGL31-LF*), *CONSTANS-LIKE 4* (*BcCOL4-MF2*), *JUMONJI DOMAIN-CONTAINING PROTEIN 18* (*BcJMJ18-LF*), *SUPPRESSOR OF FRIGIDA 4* (*BcSUF4-LF*), and *LATE MERISTEM IDENTITY 1* (*BcLMI1-LF*) (Fig. 1A; Table S7).

### GWAS of bolting time in *B. campestris*

**Figure 2:**
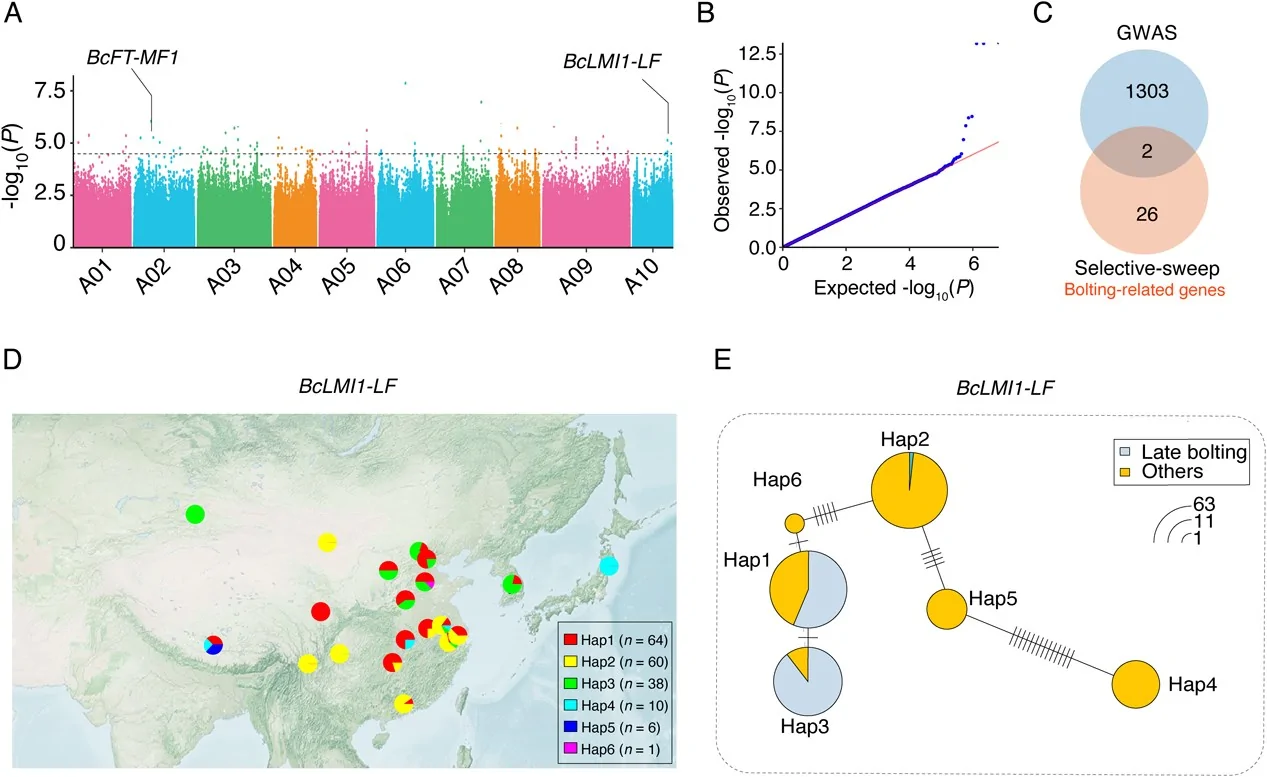
GWAS of bolting time and haplotype analysis of *BcLMI1-LF.* (A) Manhattan plot of GWAS for bolting time in *B. campestris* (*n* = 365 accessions). The horizontal dashed line indicates the threshold (−log10(*P* value) = 4.69). A01–A10 represent the ten chromosomes of *B. campestris*. (B) Q-Q plot of GWAS. (C) Venn diagram of genes identified by GWAS and bolting-related genes in selected regions during domestication. (D) Geographic distribution of *BcLMI1-LF* haplotypes across *B. campestris* accessions in East Asia, color-coded by haplotype group (Hap1–Hap6). The geographic map was adapted from NASA (https://visibleearth.nasa.gov/images/147190/explorer-base-map/147191w/). (E) Haplotype network of *BcLMI1-LF*, with circle size indicating haplotype frequency. Blue represent late-bolting haplotypes, while orange indicate other phenotypes. Numbers of short lines on the connecting lines denote mutation steps between haplotypes.

To identify genetic loci associated with bolting time in *B. campestris*, we conducted a genome-wide association study (GWAS) using bolting time of 365 accessions with 1 672 478 single nucleotide polymorphism (SNPs) (Tables S1; Fig. 2A and B). We identified 1305 genes that associate with bolting time (Fig. 2C; Table S8). GO enrichment indicated that these genes are mainly related to ‘reproductive process’, ‘shoot system development’, ‘flower development’, ‘reproductive shoot system development’ (Table S9), these terms contain about 12 key genes (Table S10). By intersecting the GWAS-significant loci with the candidate genes identified from the selective-sweep analysis (bolting-related GO terms), we narrowed the focus to only two core genes: *BcFT-MF1* and *BcLMI1-LF*. Both genes exhibit strong selection signals and significant associations with bolting time across the population. (Figs 1 and 2C; Table S10).

While *BcFT-MF1* is a well-characterized master regulator of the floral transition across species, the potential role of the HD-ZIP I member *BcLMI1-LF* in modulating bolting time during domestication prompted us to prioritize this locus for further characterization. Therefore, we performed haplotype analysis to explore its specific contribution to bolting control and potential role in *B. campestris* domestication. Haplotype analysis revealed that haplotype 3 (hap3) of *BcLMI1-LF* was predominantly distributed in the areas north of the Yangtze River (Fig. 2D and Fig. S2) and was primarily associated with the late-bolting phenotype (Fig. 2E). Therefore, we detected the expression level of *BcLMI1-LF* in the accession of haplotype 3 and found it showed significant differential expression between rosette leaf and floral meristem (Fig. S3). This expression pattern suggests that *BcLMI1-LF-Hap3* may play a pivotal role in regulating bolting-related developmental processes. Based on these findings, *BcLMI1-LF-Hap3* was selected for further function identification.

### Functional validation of *BcLMI1-LF-Hap3* in regulating bolting time

**Figure 3:**
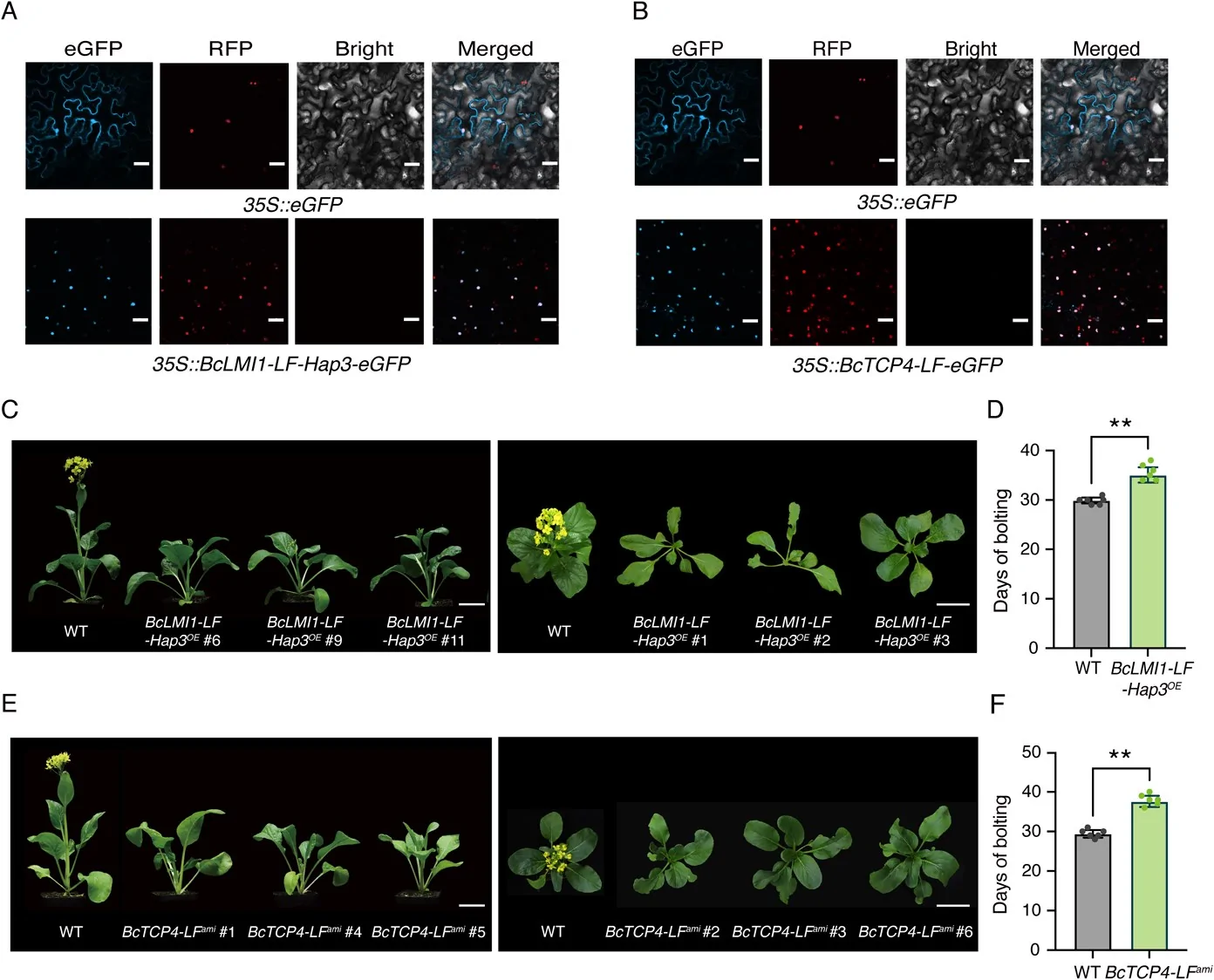
Functional validation of *BcLMI1-LF-Hap3* and *BcTCP4-LF*. (A) Subcellular localization of BcLMI1-LF-Hap3 in *Nicotiana benthamiana* leaf epidermal cells. Fluorescence from enhanced GFP and an RFP nuclear marker was merged with bright-field images. Scale bar = 50 μm. *35S::eGFP* was used as the control. (B) Subcellular localization of BcTCP4-LF in *N. benthamiana* leaf epidermal cells. Scale bar = 50 μm. *35S::eGFP* was used as the control. For ease of comparison, the *35S::eGFP* of panels A and B used the same photos. (C) Bolting and leaf phenotypes of WT and representative independent *BcLMI1-LF-Hap3^OE^* transgenic lines. Scale bar = 4 cm. (D) Days to bolting in WT and *BcLMI1-LF-Hap3^OE^* plants, ^**^  *P* < 0.01, Student’s *t*-test. Each data point represents a distinct independent transgenic line. Data are presented as mean ± SD (*n* = 6). (E) Bolting and leaf phenotypes of WT and representative independent *BcTCP4-LF^ami^* lines. Scale bar = 4 cm. (F) Days to bolting in WT and *BcTCP4-LF^ami^* lines, ^**^  *P* < 0.01, Student’s *t*-test. Each data point represents a distinct independent transgenic line. Data are shown as mean ± SD (*n* = 6).

To experimentally validate the function of *BcLMI1-LF-Hap3* in regulating bolting time, we first compared its expression levels between late- and early-bolting accessions. The expression level of *BcLMI1-LF-Hap3* was significantly higher in late-bolting accessions than in early-bolting ones (Fig. S4). We then determined the subcellular localization of BcLMI1-LF-Hap3 in *Nicotiana benthamiana*, which revealed nuclear localization, consistent with its role as a TF (Fig. 3A). Overexpression of *BcLMI1-LF-Hap3* in *B. campestris* (*BcLMI1-LF-Hap3^OE^*) caused a significant delay in bolting time compared to wild-type (WT) plants under identical conditions and also produced a visible leaf lobe phenotype varying degrees (Fig. 3C and D). This finding demonstrates that *BcLMI1-LF-Hap3* functionally regulates bolting time and leaf morphology in *B. campestris*.

To investigate how BcLMI1-LF-Hap3 regulates bolting time, we analyzed expression patterns of reproductive transition-related genes in *BcLMI1*-*LF*-*Hap3* overexpression transgenic lines via RT-qPCR. Notably, *BcTCP4-LF*, whose orthologous genes in other plants are known to control bolting time [26], showed significant down-regulation in the transgenic plants (Fig. S5). Given the shared roles of TCP4 and LMI1 in developmental transitions, we hypothesize that *TCP4* may act downstream of LMI1 to coordinate bolting time. To test this hypothesis, we first performed RT-qPCR to measure *BcTCP4-LF* expression levels in different accessions. The result showed that the expression level of *BcTCP4-LF* was significantly lower in late-bolting accessions compared to early-bolting ones (Fig. S6).

The subcellular localization results also revealed that *BcTCP4-LF* is localized in the nucleus, consistent with *BcLMI1-LF-Hap3* (Fig. 3B). To verify the role of *BcTCP4-LF* in bolting time regulation in *B. campestris*, we suppressed its expression using an artificial microRNA approach (Fig. 3E). Notably, these *BcTCP4-LF^ami^* plants displayed a phenotype similar to that of *BcLMI1-LF-Hap3^OE^* plants, exhibiting significantly delayed bolting time and different degrees of leaf lobing compared to WT plants (Fig. 3E and F). This phenotypic similarity supports the hypothesis that BcLMI1-LF-Hap3 and BcTCP4-LF function together in a hierarchical bolting-regulatory network.

### BcLMI1-LF-Hap3 directly represses *BcTCP4-LF* to regulate bolting in *B. campestris*

**Figure 4:**
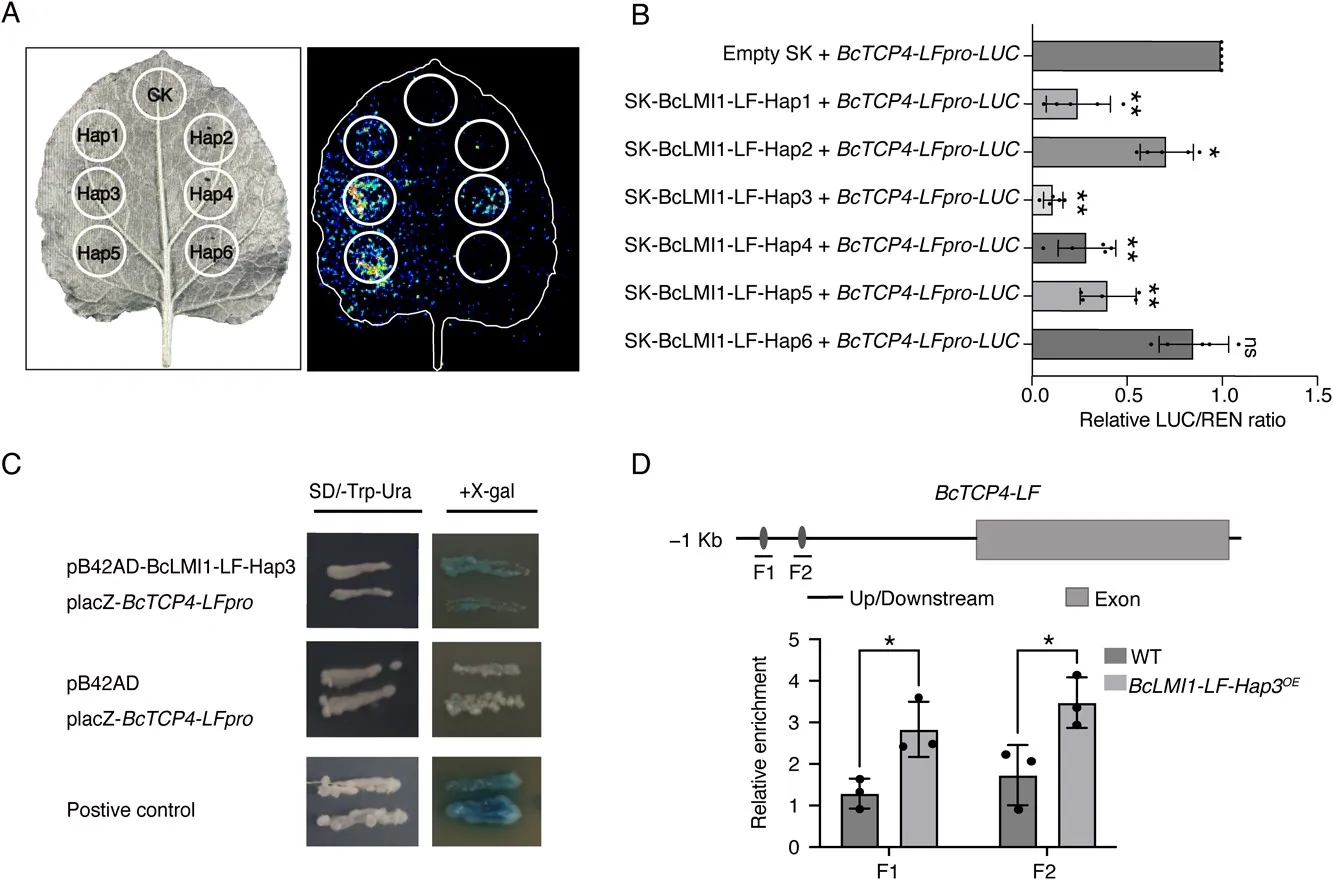
Detection of the binding of different haplotypes of BcLMI1-LF on *BcTCP4-LF* promoters. (A) Luciferase bioluminescence imaging of control (CK, empty pGreenII-62-SK (SK) vector) and six haplotypes of *BcLMI1-LF*. (B) LUC/REN ratio in *N*. *benthamiana* leaves. Error bars represent means ± SDs (*n* = 5). Asterisks indicate a statistically significant difference (^*^P < 0.05, ^**^P < 0.01) as determined by Student’s *t*-test, ns: no significance. (C) Y1H assays show that BcLMI1-LF-Hap3 (pB42AD-BcLMI1-LF-Hap3) binds to the *BcTCP4-LF* promoter (placZ-*BcTCP4-LFpro*). Blue colonies on X-gal-supplemented medium indicate a positive interaction. (D) ChIP-qPCR analysis showing enrichment of BcLMI1-LF-Hap3 at two regions (F1 and F2) of the *BcTCP4-LF* promoter in *BcLMI1-LF*-Hap3*^OE^* plants compared to WT (^*^  *P* < 0.05, Student’s *t*-test). Bars represent mean ± SD (*n* = 3).

Given that both *BcLMI1-LF-Hap3* and *BcTCP4-LF* are nuclear-localized TFs (Fig. 3A, 3B), we next investigated whether *BcLMI1-LF-Hap3* binds to the *BcTCP4-LF* promoter to repress its transcription. First, we detected the binding of six haplotypes of BcLMI1-LF on the *BcTCP4-LF* promoter. It was found that hap1, 3, 4, and 5 had obvious fluorescence signals compared with the control (Fig. 4A), while the LUC/REN ratio of BcLMI1-LF-Hap3 with *BcTCP4-LFpro* was the lowest (Fig. 4B), indicating that *BcLMI1-LF-Hap3* represses *BcTCP4-LF* expression. Sequence and domain analysis revealed that the six haplotypes differ in both their upstream regulatory regions and coding sequences (CDS), with Hap3 uniquely harboring a divergent C-terminal sequence at the protein level likely resulting from a frameshift, while all haplotypes retain an intact HOX HD (Fig. S7). Therefore, we conduct further verification through *in vivo* and *in vitro* experiments. Yeast one-hybrid (Y1H) assays confirmed a physical binding of BcLMI1-LF-Hap3 on the *BcTCP4-LF* promoter (Fig. 4C). Bioinformatic analysis identified a putative LMI1 binding motif in the upstream region of the *BcTCP4-LF* gene. To validate this interaction *in vivo*, we performed chromatin immunoprecipitation followed by quantitative PCR (ChIP-qPCR) using *BcLMI1-LF-Hap3^OE^* and WT plants. The results revealed significant enrichment of *BcTCP4-LF* promoter fragments in *BcLMI1-LF-Hap3^OE^* plants compared to WT plants (Fig. 4D), further confirming direct *in vivo* binding of BcLMI1-LF*-Hap3* to the *BcTCP4-LF* promoter.

## Discussion

Our study delineates the evolutionary trajectory and functional specialization of HD-ZIP TFs in *B*. *campestris*, uncovering their pivotal role in modulating bolting time variation during domestication. By integrating multiple analyses, we reveal how whole-genome duplication events generated genetic novelty, particularly through retention of the BcLMI1-LF paralog, which was subsequently co-opted by selection to establish haplotype-specific regulatory networks governing *BcTCP4-LF* expression. This mechanistic elucidation of the BcLMI1-LF–BcTCP4-LF regulatory axis not only provides evolutionary insights into Brassica diversification but also delivers actionable targets for precision breeding of bolting-related traits. In addition to delayed bolting, *BcLMI1-LF-Hap3^OE^* lines also display a leaf lobe phenotype, consistent with the well-established role of LMI1 homologs in regulating leaf shape in Brassicaceae. This dual phenotype suggests that BcLMI1-LF-Hap3 functions as a pleiotropic regulator of both developmental timing and leaf morphology in *B. campestris*.

The selective-sweep regions also harbor several well-characterized flowering-time and bolting regulators, including *BcFT-MF1*, *BcFD-MF2*, *BcSPL3-LF*, GA pathway components (*BcGA20OX* family and *BcGID1C-LF*), and additional factors such as *BcAGL31-LF*, *BcCOL4-MF2*, *BcJMJ18-LF*, and *BcSUF4-LF* (Fig. 1A). The co-occurrence of GA biosynthesis and signaling genes alongside transcriptional regulators within these swept loci suggests that artificial selection acted on multiple convergent pathways, consistent with the polygenic nature of bolting time as a domestication trait.

Notably, *BcTCP4-LF* itself does not fall within the selective-sweep or GWAS candidate regions, which is consistent with the view that artificial selection preferentially targets upstream regulatory genes rather than their downstream effectors. Modifying the activity of a transcriptional regulator, such as BcLMI1-LF, provides an efficient means of reshaping the downstream network while minimizing pleiotropic costs. *BcTCP4-LF* was identified as a downstream target through screening of *BcLMI1-LF-Hap3^OE^* lines at transcriptional level, in which it was consistently and significantly down-regulated. This approach using population-level selection signals to prioritize candidate regulators, then dissecting their downstream networks through functional genomics, illustrates how combining evolutionary and molecular analyses can reveal regulatory hierarchies underlying domestication traits.

Sequence comparison among the six *BcLMI1-LF* haplotypes revealed that their differentiation is collectively shaped by variations in both the upstream regulatory regions and the CDS. Among Hap1, 2, 4, 5, and 6, which share identical protein sequences, haplotype-specific upstream SNPs and indels likely account for their differential suppression of *BcTCP4-LF* promoter activity (Fig. S7A). Hap3, however, additionally harbors a divergent C-terminal sequence at the protein level (Fig. S7B), probably resulting from a frameshift mutation, which may alter its protein–protein interactions or transactivation capacity and thereby underlie its more pronounced regulatory effects. Despite this divergence, all haplotypes retain an intact HOX HD (Fig. S7C), indicating that functional differences arise primarily from C-terminal variation and upstream regulatory divergence.

**Figure 5:**
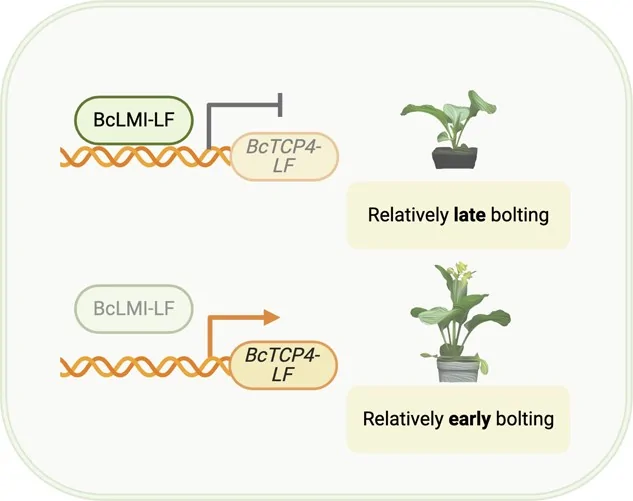
Schematic model of the BcLMI1–*BcTCP4* regulatory pathway involved in bolting time in *B. campestris*. BcLMI1-LF represses *BcTCP4-LF* expression by directly binding to its promoter, thereby delaying bolting. When this repression is released, *BcTCP4-LF* is upregulated, leading to relatively early bolting.

Strong selection signals at the *BcLMI1-LF* locus provide compelling evidence of its central role in bolting domestication. This contrasts with *HD-ZIP* I genes in cereals, where selection typically favors stress tolerance rather than developmental timing [27, 28]. A key finding of our study is the identification of BcLMI1-LF, a class I HD-ZIP TF, as a key regulator of bolting through repression of *BcTCP4-LF* (Fig. 4). This reveals a previously unrecognized cross-family relationship integrating HD-ZIP and TCP TFs (Fig. 5). While HD-ZIP III genes are well documented for their roles in vascular development via miRNA-mediated feedback loops [29–31], the functional divergence of class I HD-ZIP genes into reproductive timing regulation represents a mechanistic innovation.

The suppression of *BcTCP4-LF* by BcLMI1-LF challenges the conventional view of TCP as the primary regulators of flowering time (Figs 3 and 4). Instead, it suggests a hierarchical regulatory network in which HD-ZIP genes act upstream, potentially interfacing with hormonal signaling pathways. For example, homologs of class I HD-ZIP genes in maize and cucumber have been implicated in abscisic acid and GA responses [32, 33], raising the possibility that *BcLMI1-LF* integrates photoperiod and hormonal cues to regulate the transition from vegetative to reproductive growth. This hypothesis is supported by the observed down-regulation of *BcTCP4-LF* in *BcLMI1-LF* overexpression lines (Fig. S5), as *TCPs* are known to mediate GA biosynthesis and signaling in *A. thaliana* [34, 35]. However, it is also plausible that BcLMI1-LF functions as a broader regulatory hub, acting in parallel to its repression of *BcTCP4-LF* to directly control other key genes in the flowering time pathway. For instance, HD-ZIP I class TFs, such as HB13, negatively regulate Citrus flowering through binding to the *FLC* promoter [36]. Future genome-wide studies, such as ChIP-seq using our *BcLMI1-LF-Hap3^OE^* lines, will be critical for mapping the complete cistrome of BcLMI1-LF and building a more comprehensive model of its role in the vegetative-to-reproductive phase transition.

Consistent with the established role of *LMI1* orthologues in leaf margin development, *BcLMI1-LF-Hap3* overexpression also enhanced leaf lobing in transgenic lines (Fig. 3C and E). Notably, suppression of *BcTCP4-LF* recapitulated both the delayed bolting and the leaf lobing phenotypes observed in *BcLMI1-LF-Hap3^OE^* plants, suggesting that *BcTCP4-LF* acts as a shared downstream effector mediating both functions. This pleiotropy may therefore reflect the dual role of the BcLMI1-LF–BcTCP4-LF regulatory axis in coordinating developmental timing and leaf morphogenesis.

Nevertheless, several important questions remain. While our findings strongly support a relationship where BcLMI1-LF represses *BcTCP4-LF*, we acknowledge that definitive genetic proof requires analysis in a null mutant background. To this end, future work will focus on generating *bclmi1-lf* knockout lines using a suitable CRISPR/Cas9 system to perform complementation experiments with the different haplotypes. This will conclusively validate their functional divergence. Further exploration is also needed to understand the functional redundancy among HD-ZIP subfamilies, the role of epigenetic modifications, and the interactions between BcLMI1-LF and other key flowering regulators, all of which will help construct a more comprehensive regulatory framework.

## Conclusion‌

In summary, this study advances our understanding of the evolutionary dynamics of the HD-ZIP gene family and its pivotal contribution to bolting time domestication in *B. campestris*. Through systematic dissection of the LMI1–TCP4 regulatory axis, we establish a mechanistic paradigm whereby genome duplication events created genetic diversity that was subsequently refined through subfunctionalization and artificial selection to shape agronomically important traits. These findings reveal the evolutionary principles governing gene family expansion and functional diversification in *B. campestris*, while simultaneously establishing a molecular roadmap for targeted genetic improvement of Brassica crops.

## Materials and methods

### Plant materials and growth conditions

A diverse population of 365 *B. campestris* accessions was employed for both selective-sweep analysis and GWAS. All accessions were cultivated under identical environmental conditions at the experimental farm of the Zijingang Campus, Zhejiang University, in Hangzhou, China. Seeds were sown simultaneously on September 30th, 2023, and plants were managed according to standard agronomic practices. Bolting time was quantified as the number of days from the date of sowing to the stage when the first floral bud became visible (~1 cm in height). Phenotypic data were collected across 30 biological replicates for each accession. Based on the intrinsic responses under these uniform conditions, we defined accessions with a bolting time of less than 80 days as the ‘early-bolting group’, while those with a bolting time greater than 150 days were defined as the ‘late-bolting group’ for selective-sweep analysis. Samples were collected, immediately frozen in liquid nitrogen, and stored at −75°C for further analysis. Transgenic plants were grown under environmentally controlled conditions with a 16 h light/8 h dark photoperiod at 22°C during the day and 18°C at night, with approximately 60% relative humidity. *Nicotiana benthamiana* plants were cultivated at 25 ± 2°C under a 16-h light/8-h dark photoperiod.

### Selective-sweep analysis

VCFtools was used to calculate nucleotide diversity (*π*) and the population differentiation index (*F*_ST_) [37]. *F*_ST_ values were estimated using a 50-kb sliding window with a 5-kb step across each chromosome. Genome-wide average *F*_ST_ was calculated across population groups. The top 5% of *F*_ST_ values were considered as putative selective regions. Overlapping high-scoring windows were merged and defined as highly diverged regions between groups. Regions under selection were further analyzed using XP-CLR (https://github.com/hardingnj/xpclr) with the parameters: —size 50 000, —step 5000, —maxsnps 200, and —minsnps 5. The top 5% of regions with the highest XP-CLR scores were defined as candidate-selective-sweep regions. GO enrichment analysis was performed using TBtools (v2.0) software [38, 39]. GO terms with a corrected *P* value less than 0.05 were regarded as significantly enriched.

### GWAS analysis for the bolting trait

The chromosomal positions of the SNPs were referred to in the *B. campestris* v3.5 reference genome. SNPs were filtered (minor allele frequency > 0.05 and missing rate < 0.1) and used in the GWAS analysis for the bolting trait. Association analysis was performed using BLINK in the GAPIT 3.0 software package [40]. The Manhattan and Q-Q plots were created with the R package qqman [41]. Population structure was analyzed using ADMIXTURE (v.1.3.0) software for *K* values ranging from 2 to 10. Haplotype analysis was performed using the geneHapR [42] package of R software.

### RNA extraction and RT-PCR

Total RNA was extracted from the following materials: for early- and late-bolting accessions, shoot apex tissues were collected about to bolting; for transgenic plants, the youngest fully expanded rosette leaves were collected at approximately 30 days after sowing. All extractions were performed using TRIzol reagent (AG RNAex Pro Reagent, Cat: AG21102). First-strand cDNA synthesis was carried out using 1 μg of total RNA with the HiScript III RT SuperMix for qPCR kit (HiScript III RT SuperMIX for qPCR, Cat: R323-01, Vazyme, Nanjing, China). Quantitative real-time PCR (RT-qPCR) was performed using SYBR Green Pro Taq HS (Cat: AG11701, Accurate Biology, Changsha, China). Relative gene expression levels were calculated using the 2^−ΔΔCt^ method [43]. Gene-specific primers used for RT-qPCR are listed in Table S11.

### Sub-cellular localization

The full-length coding sequences (CDSs) of *BcLMI1-LF-Hap3* and *BcTCP4-LF* were amplified and inserted into the pFGC-eGFP vector to generate the fusion constructs *35S::BcLMI1-LF-Hap3-eGFP* and *35S::BcTCP4-LF-eGFP*. These constructs were used for transient expression assays in *N. benthamiana* leaves, co-infiltrated with a nuclear-localized red fluorescent protein (RFP) marker. The *35S::eGFP* vector served as a negative control. Green fluorescent protein (GFP) signals were detected 48 h post-infiltration using a confocal laser scanning microscope (TCS SP8-SE; Leica, Wetzlar, Germany) with excitation at 488 nm and emission at 505 to 530 nm.

### Vector construction and plant transformation

The full-length sequence of *BcLMI1-LF-Hap3* was amplified from genomic DNA using gene-specific primers (Table S11). The PCR products were cloned into the pFGC1008 vector under the control of the CaMV 35S promoter. An artificial microRNA (amiRNA) targeting *BcTCP4-LF*, based on the *MIR164* backbone, was synthesized by Beijing Tsingke Biotech Co., Ltd (Beijing, China) and cloned into the pFGC1008 vector. Recombinant vectors were introduced into *Agrobacterium tumefaciens* strain GV3101 (CAT#: AC1001, WeiDi, Shanghai, China) and subsequently transformed into ‘Guangliang caixin’ via the floral dip method [44].

### Luciferase bioluminescence imaging and dual-luciferase assays

The full-length gene sequences containing upstream region of six haplotypes of *BcLMI1-LF* were amplified and cloned into the pGreenII 62-SK vector (Promega, Madison, WI, USA). Promoter fragments of *BcTCP4-LF* were cloned into the pGreenII 0800-LUC vector (Promega, Madison, WI, USA). The effector and reporter plasmids were co-transformed into *Agrobacterium tumefaciens* GV3101 (pSoup-p19) (CAT#: AC1003, WeiDi, Shanghai, China). Experimental and control constructs were infiltrated into the same *N. benthamiana* leaf. The infiltrated plants were incubated in the dark at 25°C for 36 h. The D-Luciferin potassium salt (CAT#: ST196, Beyotime, Shanghai, China) was used in the luciferase bioluminescence imaging assay. The activated luciferase reconstitution signals were imaged using the BioRad chemiluminescence image analysis system (California, USA). Luciferase activity was measured using the Dual-Luciferase Reporter Assay System (Promega, Madison, WI, USA) according to the manufacturer's instructions. The transcriptional activity was calculated as the ratio of Luciferase/Renilla (LUC/REN).

### Yeast one-hybrid assays

Promoter fragments (~1 kb) of *BcTCP4-LF* were PCR-amplified using gene-specific primers (Table S11) and cloned into the pLacZ vector (Promega, Madison, WI, USA) as bait constructs. The full-length CDS of *BcLMI1-LF* was inserted into the pB42AD vector to serve as prey. Y1H assays were performed using the MATCHMAKER system (Clontech, Mountain View, CA, USA) according to the manufacturer's protocol. Bait plasmids were transformed into Saccharomyces cerevisiae strain EGY48 (Cat#: YC1030, WeiDi, Shanghai, China) and selected on SD/-Trp medium (Cat#: 630308, Clontech, USA). Protein–DNA interaction was determined based on the growth ability of the co-transformed yeast cells on the SD/-Trp/-Ura (DO Supplement-Trp/-Ura, CAT#: PM2260, Coolaber, Beijing, China) dropout medium containing X-gal (X-gal CAT#: CX11921, Coolaber, Beijing, China) at 30°C. Empty pB42AD and pLacZ served as negative controls.

### ChIP-qPCR

Leaves from 30-day-old transgenic plants expressing *35S::BcLMI1-LF-Hap3^OE^-3 × HA* were collected in crosslinking buffer with 1% formaldehyde for 10 min under vacuum, followed by quenching with 0.125 M glycine for 5 min. After three washes with distilled water, the cross-linked tissues were immediately frozen in liquid nitrogen. Chromatin immunoprecipitation was performed using the EpiQuik Plant ChIP Kit (EPIGENTEK ANNORON, CAT#: P-2014-24, Beijing, China) and anti-HA (HA-Tag (C29F4) Rabbit mAb, Cell Signaling Technology, Danvers, MA, USA). Primers were designed based on the predicted binding motifs by using the JASPAR website [45] (Table S11). RT-qPCR was performed according to the protocol described previously (SYBR Green Pro Taq HS, Cat: AG11701, Accurate Biology, Changsha, China).

### Statistical analysis

GraphPad Prism v10.2.1 (GraphPad Software, San Diego, USA) was used for statistical analysis.

## Supplementary Material

Web_Material_uhag157

## Data Availability

All data from this study are included in the submitted article.
