# Supplementary material for: The HD-ZIP I transcription factor BcLMI1 regulates bolting time in Brassica campestris by repressing BcTCP4
Source: Hortic Res. 2026 Apr 17;13(9):uhag157. doi: 10.1093/hr/uhag157 (PMC13522724; doi:10.1093/hr/uhag157)
Supplement: Supplementary file 1 [file web_material_uhag157.zip › Supplementary Figures.pdf]

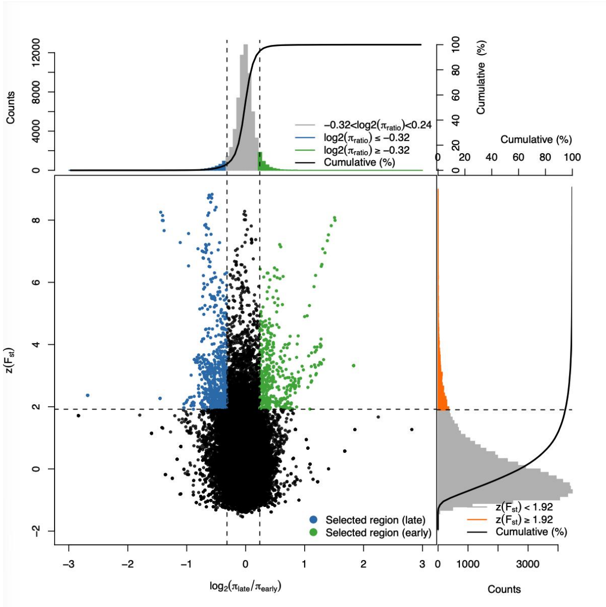

Fig. S1 Joint analysis of F_{ST} and π for identifying selection signals associated with the bolting time in the *B. campestris* genome.

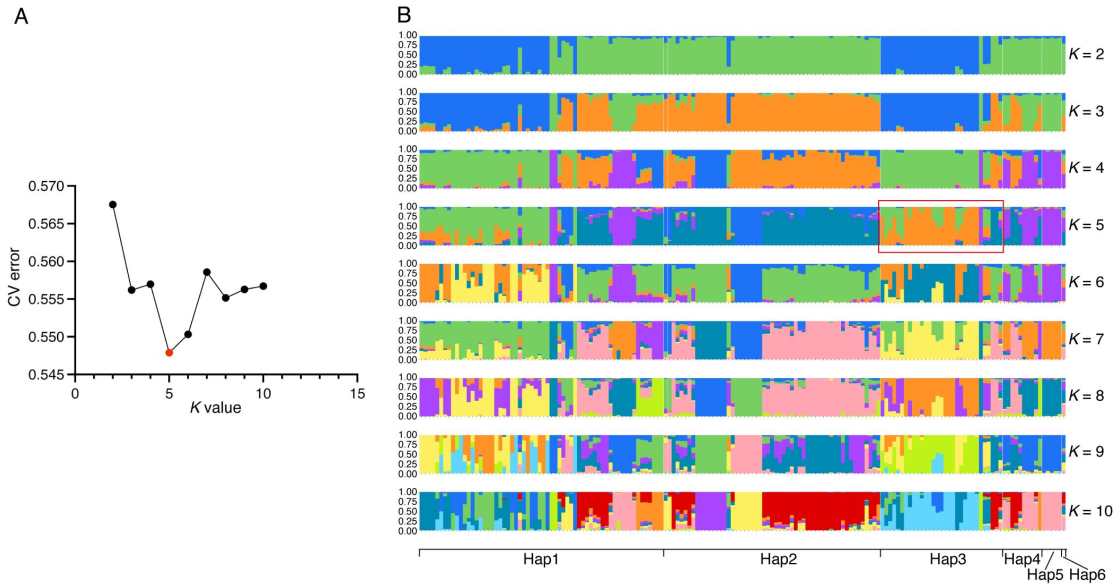

Fig. S2 Population structure of *Brassica campestris* accessions. (a) Cross-validation errors for *K* from 2 to 10, the red circle represents the lowest CV error value. (b) Model-based clustering analysis of ancestry kinship (*K* = 2-10).

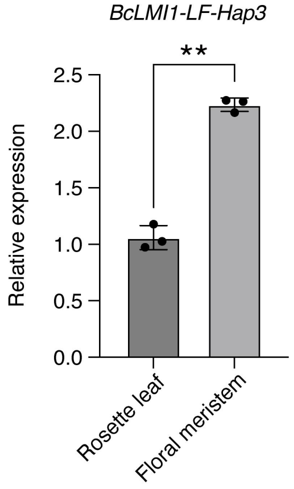

Fig. S3 Relative expression of *BcLMI1-LF-Hap3* in rosette leaf and floral meristem. Data are presented as mean ± SD (n = 3). ** indicates a statistically significant difference ($p < 0.01$) as determined by Student's t-test.

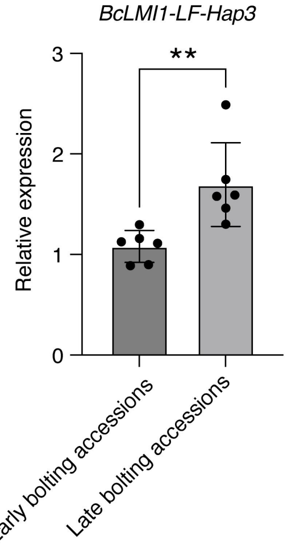

Fig. S4 Relative expression of *BcLMI1-LF-Hap3* in early- and late-bolting accessions. RNA was extracted from shoot apex tissues about to bolting. Data are presented as mean ± SD (n = 6, accessions). ** indicates a statistically significant difference ($p < 0.01$) as determined by Student's t-test.

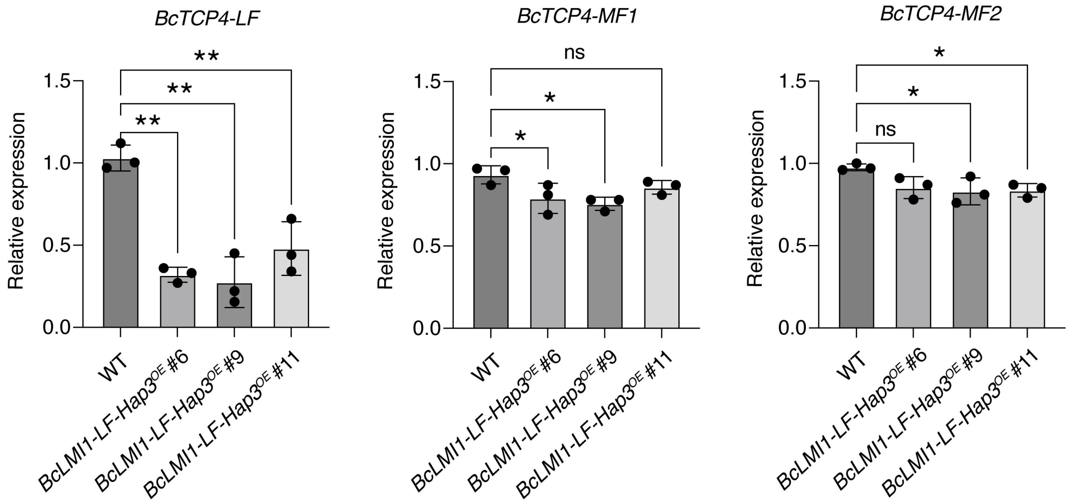

Fig. S5 Relative expression level of three copies of *BcTCP4* in *BcLMI1-LF-Hap3*^{OE} transgenic plants. RNA was extracted from the youngest fully expanded rosette leaves of plants at approximately 30 days after sowing. Data are presented as mean ± SD ($n = 3$). * $p < 0.05$, ** $p < 0.01$, one-way ANOVA Dunnett's test. LF: least fractioned, MF1: medium fractioned, MF2: most fractioned.

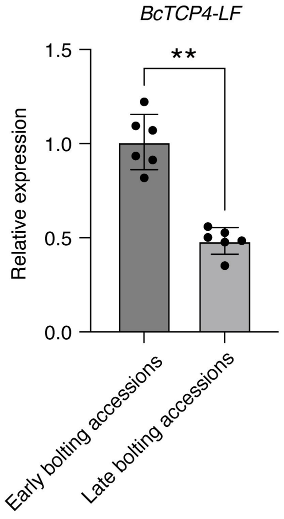

Fig. S6 Relative expression of *BcTCP4-LF* in early- and late-bolting accessions. RNA was extracted from shoot apex tissues about to bolting. Data are presented as mean ± SD (n = 6, accessions). ** indicates a statistically significant difference (p < 0.01) as determined by Student's t-test.

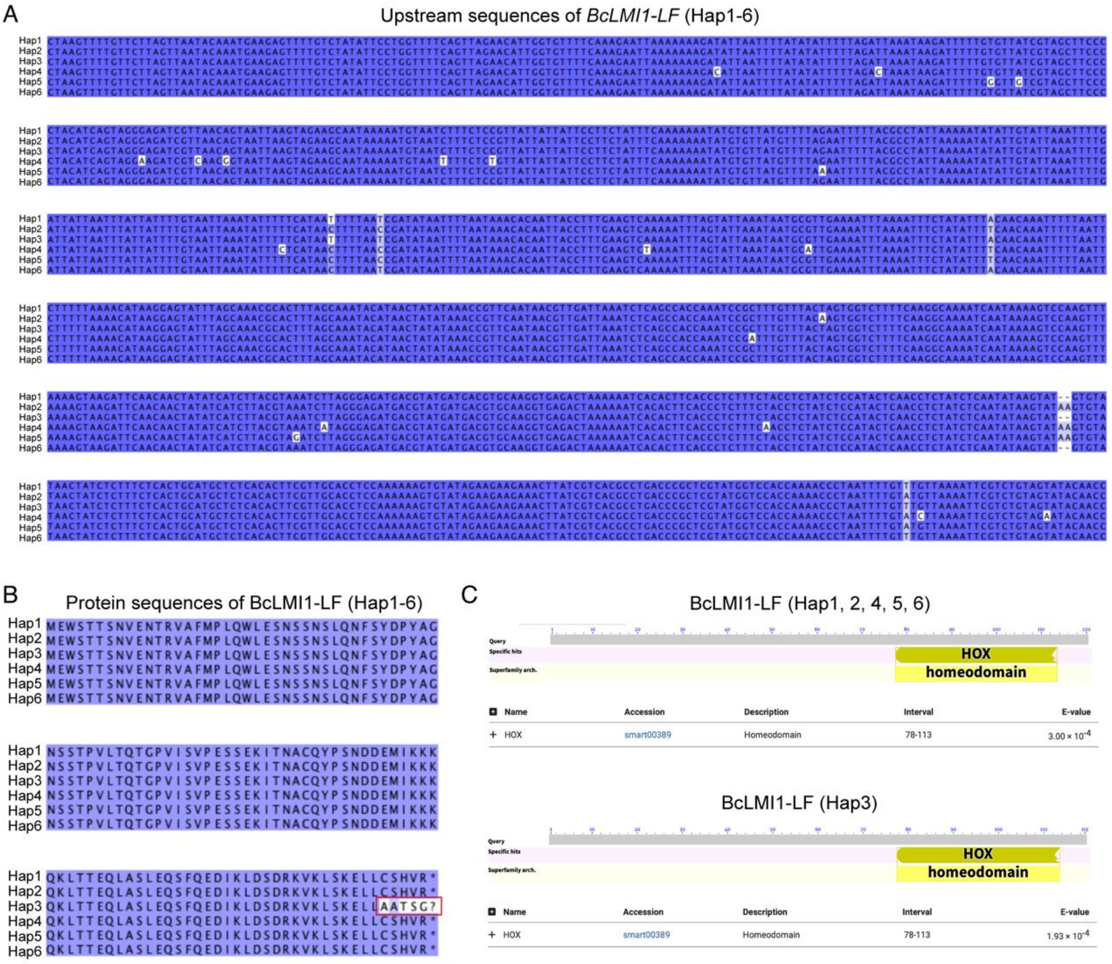

Fig. S7 Sequence and domain comparison of *BcLMI1-LF* across six haplotypes. **(A)** Multiple sequence alignment of the upstream regulatory regions of *BcLMI1-LF* in Hap1–6. **(B)** Multiple sequence alignment of the deduced protein sequences of BcLMI1-LF in Hap1–6. The divergent C-terminal sequence of Hap3 is boxed in red. **(C)** Domain analysis of BcLMI1-LF protein sequences.
